# Finding Your Seat at the Table: Roles for Librarians on In-stitutional Regulatory Boards and Com-mittees

**DOI:** 10.5195/jmla.2022.1559

**Published:** 2022-07-01

**Authors:** Tyler Moses

**Affiliations:** 1 TMoses@novanthealth.org, Library Services, Presbyterian Medical Center, Novant Health, Inc., Charlotte, NC.

In *Finding Your Seat at the Table*, editors Susan Harnett and Laureen Cantwell illustrate how librarians can support Institutional Review Boards (IRB) and Institutional Animal Care and Use Committees (IACUC) at their respective institutions. Institutional Review Boards are committees charged with reviewing and monitoring research using human subjects whereas Institutional Animal Care and Use Committees oversee the care and management of animals used for labs or experiments. “Part I: Institutional Review Board” describes the role and function of IRBs, major historical events that lead to the creation of the board, and how librarians can support IRBs at their own institution. Similarly, “Part 2: Institutional Animal Care and Use Committee” outlines the role of IACUC, a synopsis of the history of animal research, and how librarians can assist in animal research. “Part III: Creating and Expanding Opportunities for Libraries and Librarians in Institutional Research,” provides case studies of how librarians can support institutional human and animal research.

The authors did an excellent job explaining the review processes of both IRB and IACUC. For instance, chapter one “An Introduction to Institutional Review Board”, differentiates the types of review processes (expedited review versus full committee review) that researchers can undergo based on the activities of their studies. Chapter four “The Institutional Animal Care and Use Committee” explains the different roles members on the committee perform and how they contribute to the review process. The biggest strength of the book is the myriad of examples of how libraries can support institutional research. Additionally, chapter six “Librarian and the IACUC”, goes in depth of how to conduct literature searches on animal alternatives to support protocol review for IACUC. The authors of chapter eight “The Novice Investigator” describe how librarians instruct researchers on literature searches' best practices as part of responsible conduct of research training. Another unique aspect of the book is its description of how IACUC impacts the number of procedures conducted in veterinary education. Lastly, the book lists resources readers can utilize to increase their knowledge and skills related to human and animal research.

One of the weaknesses of the book is the lack of laymen's terms used to explain the policies and ethics that govern both committees. The myriad of legal jargon in chapters two and four can make it difficult for the readers to follow how the policies and ethics impact the committees' activities. Additionally, the book primarily focuses on institutional boards at academic institutions though two case studies in chapter twelve “Expanding Opportunities for Librarians within Institutional Research Activities” describe support activities at a Naval Medical Center and a healthcare facility that is part of a hospital network.

Overall, this book serves as an excellent resource for any librarian interested in supporting institutional research.

